# The smallest eating the largest: the oldest mammalian feeding traces on dinosaur bone from the Late Jurassic of the Junggar Basin (northwestern China)

**DOI:** 10.1007/s00114-020-01688-9

**Published:** 2020-07-19

**Authors:** Felix J. Augustin, Andreas T. Matzke, Michael W. Maisch, Juliane K. Hinz, Hans-Ulrich Pfretzschner

**Affiliations:** 1 Institut für Geowissenschaften, Eberhard Karls Universität Tübingen, Hölderlinstraße 12, 72074 Tübingen, Germany; 2 Albstadt, Germany

**Keywords:** Bite marks, Early mammals, Palaeobiology, Dinosaurs, Late Jurassic, Junggar Basin

## Abstract

Reconstructing trophic interactions in ancient ecosystems is an important and fascinating branch of palaeontological research. Here we describe small bioerosional traces that are preserved on sauropod bone from the early Late Jurassic Qigu Formation (Oxfordian) of Liuhuanggou gorge in the southern Junggar Basin (Xinjiang Province, northwestern China). The most likely producers of these traces are tiny Mesozoic mammals as evinced by the small size of the traces as well as by their paired and opposed arrangement. The feeding traces are only superficially preserved on the bone surface and most likely were inflicted unintentionally during feeding. The occurrence of the bite marks along small ridges and the “gnawed” appearance of the bone surface points to selective feeding on the remaining soft tissues of the dinosaur carcass. The traces represent the oldest direct evidence for mammalian feeding behaviour in the fossil record. Additionally, these traces expand the known range of the early mammalian feeding repertoire significantly and shed light on the palaeobiology and palaeoecology of early mammals, a field that has remained evasive for a long time.

## Introduction

For more than 160 million years, mammals lived in the shadow of the dinosaurs, remaining small and elusive with an average adult body size close to 100 g (Luo 2007; Lee and Beck 2015). Nonetheless, recent discoveries demonstrate that Mesozoic mammals were ecologically diverse and occupied various ecological niches. They ranged from ground-dwelling generalists to specialists with semi-aquatic, fossorial, arboreal and even gliding habits (Luo 2007). This ecological diversity suggests an equally varied diet, probably encompassing herbivory, insectivory, carnivory and omnivory (Luo 2007). The reconstruction of the diet and feeding behaviour of Mesozoic mammals is, however, largely based on circumstantial evidence such as tooth morphology. Direct evidence for feeding behaviour is scarce and so far limited to a stomach content from the Early Cretaceous (Hu et al. 2005), and four instances of feeding traces on bones from the Late Cretaceous (Longrich and Ryan 2010; de Valais et al. 2012; Gianechini and de Valais 2015; Augustin et al. 2019).

Here we describe bioerosional traces preserved on dinosaur bone from the early Late Jurassic Qigu Formation (Oxfordian, approximately 160 Ma) of the southern Junggar Basin, northwestern China, and argue that they represent the oldest direct evidence of mammalian feeding behaviour. This predates the hitherto oldest feeding traces ascribed to mammals from the Late Cretaceous (Longrich and Ryan 2010; de Valais et al. 2012; Gianechini and de Valais 2015; Augustin et al. 2019), by more than 60 million years. Thus, our findings significantly expand our understanding of the ecology and behaviour of early mammals.

## Material and methods

### Geological setting

**Fig. 1:**
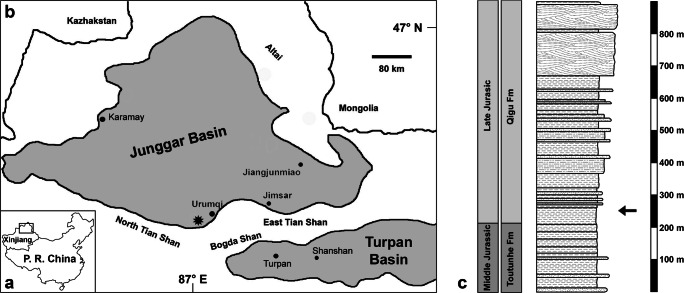
Map and stratigraphy of Liuhuanggou gorge. (a) Inset shows the position of the Junggar Basin (rectangle) within Xinjiang Province in northwestern China. Modified from Augustin et al. (in press). (b) Geographic map of the Junggar Basin (Xinjiang Province, northwest China) and the position of Liuhuanggou gorge (asterisk). (c) Stratigraphy and sedimentology of the Toutunhe and Qigu Formation at the northern flank of Liuhuanggou gorge. The arrow marks the position of the dinosaur bone-bed from which the bite marked bone was recovered. Modified from Ashraf et al. (2010)

The bioerosional traces were discovered on a bone fragment of a sauropod dinosaur at the northern flank of Liuhuanggou gorge (43° 42′ 56″ N, 87° 10′ 21″ E) in the southern Junggar Basin (Xinjiang Province, northwestern China), approximately 40 km southwest of Urumqi (Fig. 1a, b). The locality was discovered during the Sino-German-Project in 2000, a joint expedition by the University of Tübingen, the Nanjing Institute for Geology and Palaeontology and the Geological Survey No. 1 of Xinjiang. The trace-bearing bone was associated with other fragmentary bones of a large-sized mamenchisaurid sauropod dinosaur in a bone-bed horizon in the lower part of the Qigu Formation, approximately 50 m above the boundary with the underlying Toutunhe Formation (sensu Ashraf et al. 2010). The sauropod dinosaur total length was estimated to be more than 20 m. Theropod teeth were found interspersed between the sauropod remains and belong to a large-sized carnosaur and a smaller theropod of unknown affinities (Maisch and Matzke 2003).

The Qigu Formation is early Late Jurassic in age (Eberth et al. 2001; Li et al. 2014; Fang et al. 2015, 2016) and has a thickness of 680 m at Liuhuanggou gorge (Ashraf et al. 2010). It consists of massive reddish mudstones and siltstones interbedded with fine-grained sandstone horizons (Fig. 1c). The sediments of the Qigu Formation were probably deposited on a low-gradient alluvial plain, composed of extensive floodplain areas, adjacent to braided river systems and slow-flowing meandering rivers (Eberth et al. 2001; Li et al. 2014; Fang et al. 2016). Environmental conditions during the time of deposition have been interpreted as arid and highly seasonal (Ashraf et al. 2010; Li et al. 2014; Fang et al. 2015, 2016). The diverse vertebrate fauna of the Qigu Formation thus far comprises hybodont sharks, actinopterygian fishes, temnospondyl amphibians, mammals, xinjiangchelyid turtles, squamates and choristoderes, crocodylomorphs, pterosaurs, as well as sauropods, small and large theropods, stegosaurs and ankylosaurs among dinosaurs (Maisch et al. 2003, 2004a, b, 2005; Maisch and Matzke 2005, 2014, 2017; Wings et al. 2007; Schellhorn et al. 2009; Martin et al. 2010; Richter et al. 2010; Augustin et al. in press). The mammal assemblage is particularly rich and comprises five different taxa: the haramiyid *Sineleutherus*; two docodonts, *Tegotherium* and *Dsungarodon*; the stem zatherian *Nanolestes*; and an indeterminate amphilestid triconodont (Pfretzschner et al. 2005; Maisch et al. 2005; Martin et al. 2010). All of these mammals were found in a microvertebrate-bearing bone-bed at Liuhuanggou gorge close (300 m) to the dinosaur bone-bed site. Stratigraphically, the mammal site occurred only slightly higher (70 m) in the section, indicating that the dinosaur and the mammals lived more or less coeval in the same ecosystem.

### Description of sample

**Fig. 2:**
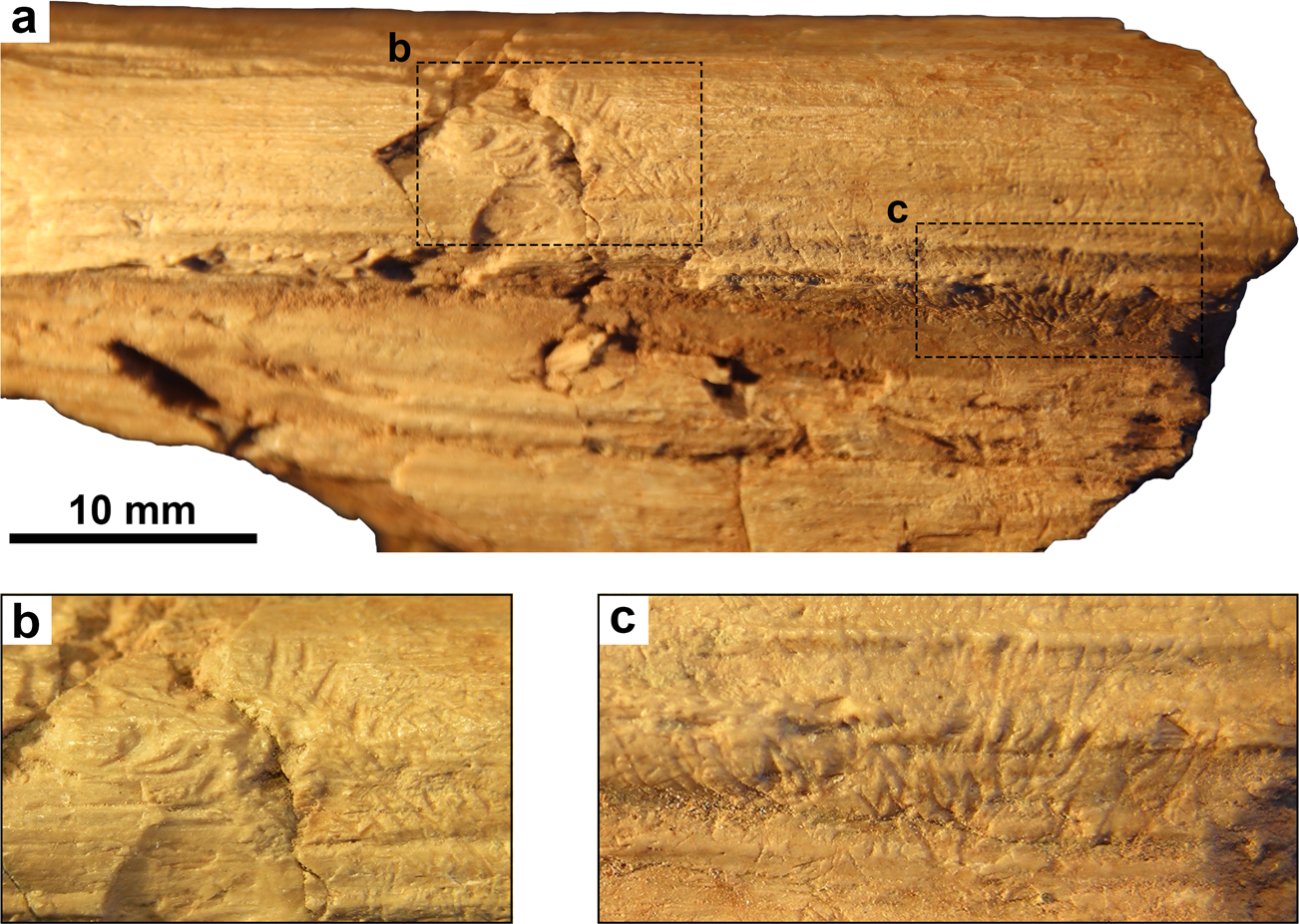
Cervical rib of a mamenchisaurid sauropod (SGP 2000/16) from the lower Qigu Formation (Oxfordian, Late Jurassic) of Liuhuanggou gorge, northwestern China. (a) Overview photograph of the specimen (SGP 2000/16) displaying bioerosional traces. (b) Detail photograph of the bioerosional traces from a slightly different angle than in (a) for better visibility of the traces. (c) Detail photograph of the bioerosional traces from a slightly different angle than in (a) for better visibility of the traces

The bioerosional traces are preserved on a fragmentary cervical rib of an indeterminate mamenchisaurid sauropod dinosaur (SGP 2000/16) (Fig. 2a). Although dozens of cervical rib fragments have been recovered from the dinosaur site at Liuhuanggou, small bioerosional traces were only found on one bone fragment. Interestingly, the traces are limited to small ridges on the bone and do not occur on the more level or concave surfaces in between (Fig. 2b, c). They appear as elongated, shallow depressions that are restricted to the cortical bone and are principally arranged in parallel pairs. The traces are usually aligned opposite to each other, with one set of traces visible on one side of the ridge and the other set on the opposite side (Fig. 2b, c). The traces have a length of approximately 0.5–1.5 mm and a width of 30–250 μm. For the measurement of the trace size, we used the methodology of Tong et al. (2008).

## Discussion

Small and elongated traces on bones have been linked to the following causes: trampling by large vertebrates (e.g. Behrensmeyer et al. 1986; Olsen and Shipman 1988; Fiorillo 1991; Augustin et al. 2019), feeding by insects (e.g. Fejfar and Kaiser 2005; Britt et al. 2008; Pomi and Tonni 2011; Backwell et al. 2012; Gianechini and de Valais 2015; Augustin et al. 2019) and feeding by vertebrates (Longrich and Ryan 2010; Gianechini and de Valais 2015; Augustin et al. 2019). Below, we discuss all the possible trace-makers for the bioerosional traces described in the previous sections of this manuscript and conclude that they were most likely produced by early Mesozoic mammals.

Trampling by large vertebrates has often been inferred by the presence of small micro-striations on bone surfaces in fossil and sub-fossil settings. These micro-striations are much smaller, parallel and more uniform to one another (Behrensmeyer et al. 1986: Figs. 2–3; Olsen and Shipman 1988: Figs. 3–4; Fernández-Jalvo and Andrews 2016: Figs. A.84–86), and are therefore different from the traces described here.

Insect traces are widespread in the fossil record and range in morphology from cavities and tunnels to star-shaped pits (Augustin et al. 2019). Elongated grooves have also been ascribed to insects, particularly dermestid beetles (e.g. Britt et al. 2008) and termites (e.g. Fejfar and Kaiser 2005; Pomi and Tonni 2011; Backwell et al. 2012; Augustin et al. 2019). The well-preserved traces from the Qigu Formation (Fig. 2b) differ from these insect feeding traces in having a teardrop shape, with one pointed and one blunt end. When several scratches occur as opposed parallel pairs, the blunt ends face each other (Fig. 2a). In these traces, the deepest part of the scratches is near the blunt end and not near the mid-length as it is the case in termite traces (Augustin et al. 2019: Fig. 7A–B). Additionally, termite feeding traces often comprise star-shaped pits and are usually superimposed on one another, resulting in heavily bioeroded surfaces lacking most of the compact bone layer (e.g. Fejfar and Kaiser 2005; Pomi and Tonni 2011; Backwell et al. 2012; Augustin et al. 2019). Therefore, we conclude that insects are not responsible for the traces.

Vertebrate feeding traces are frequently preserved on bones and usually come in the form of punctures, scores and grooves. The only vertebrates from the Qigu Formation that fall within the size range of the traces described above are mammals (Pfretzschner et al. 2005; Martin et al. 2010), as well as squamates and one possible choristoderan (Richter et al. 2010); however, the dentition pattern of squamates and choristoderans is incompatible with the arrangement of the bite marks described here. Both possess a homodont dentition with closely packed and irregularly spaced teeth in the upper and lower jaw, and therefore, they cannot be the producer of these traces.

Mammals are the only vertebrates that possess two sets of procumbent, paired incisors in the upper and lower jaw and can produce bite marks of the kind described above. The mammalian teeth recovered from Liuhuanggou mostly comprise molars and premolars that range in size from 0.5 to 1.5 mm (Martin et al. 2010). So far, only one incisor belonging to *Sineleutherus uyguricus* is known that has a cusp width of 200 μm and thus matches the size of the larger bite marks very well (Fig. 2b). For the other four taxa from this locality, unfortunately, incisors are unknown, but their estimated size lies well within the range expected from the traces. Moreover, the traces here described show all the characteristics of feeding traces ascribed to mammals from the Late Cretaceous (Longrich and Ryan 2010; de Valais et al. 2012; Gianechini and de Valais 2015; Augustin et al. 2019).

Feeding traces by extant insectivorous mammals described in the catalogue of vertebrate taphonomic identifications by Fernández-Jalvo and Andrews (2016: Figs. A.175–186) are almost identical to the ones described here, lending further support to the interpretation of mammals as trace-makers. Interestingly, the bite marks of insectivorous mammals more closely resemble the traces from Liuhuanggou than those of rodents (Andrews 1990: Fig. 1.3E–F; Fernández-Jalvo and Andrews 2016: Figs. A.187–190). This is expected because of the similarity of the dentition pattern and the reconstructed diet between extant insectivorous mammals and the mammals described from Liuhuanggou (Pfretzschner et al. 2005). Therefore, we contend that the feeding traces can be confidently assigned to mammals based on the small size of the traces and their characteristic, paired arrangement with an opposing pair of bite marks preserved on the opposite side of ridges or protuberances.

Although the traces described herein are overall similar to bioerosional traces of insects, a thorough comparison with extant insectivore bite marks and accounts from the fossil record clearly indicates a mammalian origin of the traces. In general, the interpretation of trace fossils, especially bioerosional traces, is often contentious and a matter of debate. However, we are confident that the most likely explanation of the bioerosional traces presented in this study is feeding activity by mammals based on the typical arrangement in opposed pairs of some of the traces.

Due to the extreme size discrepancy of predator and prey, the bite marks clearly represent scavenging behaviour. Scavenging behaviour is expected in early mammals because of their moderate dental complexity that allowed a generalized diet. Additionally, animal tissue provides a source for proteins, lipids and minerals that are otherwise hard to obtain for such small animals with an estimated adult size of less than 100 g. Since the feeding traces are only superficially preserved on the bone surface (Fig. 2b, c), they most likely were inflicted unintentionally during feeding. The arrangement of the bite marks along small ridges and the “gnawed” appearance of the bone surface, points to selective feeding on the remaining soft tissues of the dinosaur carcass that were still attached to the bones.

The mammalian bite marks described here represent the oldest direct evidence for a carnivorous diet in early mammals. They also represent the oldest record of scavenging behaviour. Our findings expand the known range of the early mammalian feeding repertoire significantly and shed light on the palaeobiology and palaeoecology of early mammals, a field that has been poorly known for a long time. Until now, there was a significant gap between the earliest known mammals with a generalized heterodont dentition from the Late Triassic (220 Ma) and the oldest feeding traces from the Late Cretaceous (100 Ma). This study considerably reduces this gap.

## Data Availability

The specimen is currently housed at the Palaeontological Collection of the University of Tübingen and will eventually be transferred to a Chinese public collection.
